# An earthworm-like modular soft robot for locomotion in multi-terrain environments

**DOI:** 10.1038/s41598-023-28873-w

**Published:** 2023-01-28

**Authors:** Riddhi Das, Saravana Prashanth Murali Babu, Francesco Visentin, Stefano Palagi, Barbara Mazzolai

**Affiliations:** 1 Bioinspired Soft Robotics Lab, Istituto Italiano di Tecnologia, Genoa, Italy; 2 The BioRobotics Institute, Scuola Superiore Sant’Anna, Pontedera, Italy; 3 Center for Soft Robotics, SDU Biorobotics, The Maersk Mc-Kinney Moller Institute, University of Southern Denmark, Odense, Denmark; 4 Department of Computer Science, Università degli Studi di Verona, Verona, Italy

**Keywords:** Mechanical engineering, Biomedical engineering

## Abstract

Robotic locomotion in subterranean environments is still unsolved, and it requires innovative designs and strategies to overcome the challenges of burrowing and moving in unstructured conditions with high pressure and friction at depths of a few centimeters. Inspired by antagonistic muscle contractions and constant volume coelomic chambers observed in earthworms, we designed and developed a modular soft robot based on a peristaltic soft actuator (PSA). The PSA demonstrates two active configurations from a neutral state by switching the input source between positive and negative pressure. PSA generates a longitudinal force for axial penetration and a radial force for anchorage, through bidirectional deformation of the central bellows-like structure, which demonstrates its versatility and ease of control. The performance of PSA depends on the amount and type of fluid confined in an elastomer chamber, generating different forces and displacements. The assembled robot with five PSA modules enabled to perform peristaltic locomotion in different media. The role of friction was also investigated during experimental locomotion tests by attaching passive scales like earthworm setae to the ventral side of the robot. This study proposes a new method for developing a peristaltic earthworm-like soft robot and provides a better understanding of locomotion in different environments.

## Introduction

Nature offers many examples of animals that use both the flexibility of their bodies and the ability to generate physical traveling waves along the length of their body to move and explore different environments, such as snakes^1,2^, earthworms^3^, snails^4^, and caterpillars^5,6^. Natural burrowers^7^ such as earthworms are entirely soft and possess a hydrostatic skeleton^8^. They use alternating contractions of muscle layers to propel themselves both below and above the soil surface by generating retrograde peristaltic waves^9^. The individual segments (metameres) of the worm each have a specific quantity of fluid, and perform independent, localized and variable movement patterns, due to transverse sub-divisions by muscular membranes^8,10^. The antagonistic contraction of muscle layers on these constant volume chambers, results in a shape change of each segment and the alternate generation of penetration and anchoring forces^11–14^. Below the surface, cycles of alternate subterminal radial expansion and longitudinal elongation, help in the process of burrowing by extending the anteriorly placed discoidal crack. Moreover, radially expanded regions help the organism to locally anchor itself within the surrounding medium^15^. Earthworm movement has been considered to be similar to plant root propagation^16^ and the equations governing this penetration-expansion model^17^ have also been found to be appropriate for earthworm subsurface burrowing. When crawling above the surface, the presence of hair-like protrusions^18^ on the ventral side of the worm helps to generate an anisotropic frictional force, which prevents backward slipping, and results in the forward locomotion of the worm^19^.

Inspired by such natural organisms, several robots have been developed to move/burrow in real soil or in a granular medium^20–28^. Burrowing and steering were explored using a robot^29,30^ with motor-controlled flexible units. Then, its peristaltic wave generation capability was combined with the material removal function of an auger drill further to develop it into an earthworm-inspired robot^21–24^ for planetary excavation. A robot that fabricates its own body using additive manufacturing, to move in a granular medium was developed using plant roots as a model^25^. Inspired by Polychaeta (i.e., a class of annelid worms), a robot was developed to move in a granular medium by applying the principles of local fluidization through lateral motion^26^. A very recent demonstration of the fluidization of granules used blowers to successfully dig in a sand environment^27^. Another soft robotics approach was developed to explore how incorporating a kirigami skin into an earthworm-inspired robot improves its locomotion in cohesive soil^28^. In our previous work, we attempted to identify the optimal gait pattern for a soft earthworm-inspired robot in a granular medium, by comparing different wave patterns to understand the influence of peristaltic waves in locomotion^20^. Developing a soft earthworm-inspired robot that can move in a confined space presents various challenges.

Our work demonstrates the interaction of forces and anisotropic friction between the medium and the robot. Previous studies^31^ of soft earthworm-like robots mostly intended for locomotion have demonstrated the limitations of the design of peristaltic motion by elongation or compression which can produce either a longitudinal or a radial force. The soft actuators used in most peristaltic robots switch between two configurations. They generate forces during actuation, but remain passive when released^32–34^. Combining the motion and bi-directional force generation into a single actuator system can better demonstrate the muscular behavior observed in earthworms, and thus represents the missing building block for such a system. Many actuation technologies have demonstrated similar body motions, which are activated independently or together to achieve locomotion^35–37^. Most earthworm-like soft robots have been demonstrated moving on a flat surface^36–49^ or in confined spaces such as intestinal tracts^35,50–53^ and inside pipes^32,33,47,54–60^. These robots are fabricated based on pneumatic actuators (positive and negative pressure) and other non-pneumatic technologies, such as shape memory alloys (SMAs), dielectric elastomers (DEAs), and tendon-based systems. Pneumatic systems are commonly used for actuating peristaltic soft robots^20,35,46–49^ due to their ease of fabrication, response speed, and high force generation. Artificial muscles and McKibben actuators produce peristaltic motion when actuated and are generally included as a modular unit in these robots. Gait patterns are generated by solenoid valves to control locomotion speed and the interaction with the surface. In terms of non-pneumatic systems, SMAs have been applied as an actuation technology to generate peristaltic motion in soft robots, due to their low operating noise and low actuation voltage^36,39,40,61^. Dielectric elastomers take advantage of strong deformation and shape change through electrical stimuli, and have been used to generate peristaltic motion on planar surfaces^42–44^. Tendons have been used to generate continuous waves in a worm-inspired robot, which was further developed in following iterations^37,62,63^. The wireframe-based mesh robot^62,63^ was able to control the shape of its body and generate peristaltic waves. The robot was further developed in the subsequent iterations to have accurate segment co-ordination for effectively controlling the friction to create maximum locomotion efficiency^37,64,65^. Generating locomotion gaits play a vital role in efficient peristaltic locomotion based on generic locomotion algorithms^29,66–70^. A study^66^ developed a kinematic model inspired by earthworm morphology, paving the way for the development of a gait generation algorithm based on the mechanism of peristaltic waves. A follow-up work^67^ studied gait analysis and its experimental verification with a robot. A more recent work^68^ presented a new approach for controlling the locomotion of metameric robots through phase coordination. In another study^69^ , a model for gait generation was developed considering terrestrial worms as chains of mass points with ground interactions via spikes. A continuous model developed in another study^70^ considered the worm as compressible and incompressible rod-like bodies in peristaltic locomotion.

Following the alternating metameric configuration changes found in biology, we developed a soft earthworm-like robot based on a biomimetic actuator, referred to as a peristaltic soft actuator (PSA). This actuator demonstrates antagonistic motion, like an earthworm segment with only a single actuator input. When developing the PSA, we translated this design principle to generate and transmit longitudinal and alternately radial forces. To estimate the performance of the actuator, we measured force and displacement both in the air and in a granular medium. Varying the amount and type of fluid had a significant effect on the performance of the actuator. We then performed motion experiments with the robot on a planar surface, in a granular medium at different depths, and in a pipe.

## Results

### Design translation and working principle of the peristaltic soft robot

**Figure 1:**
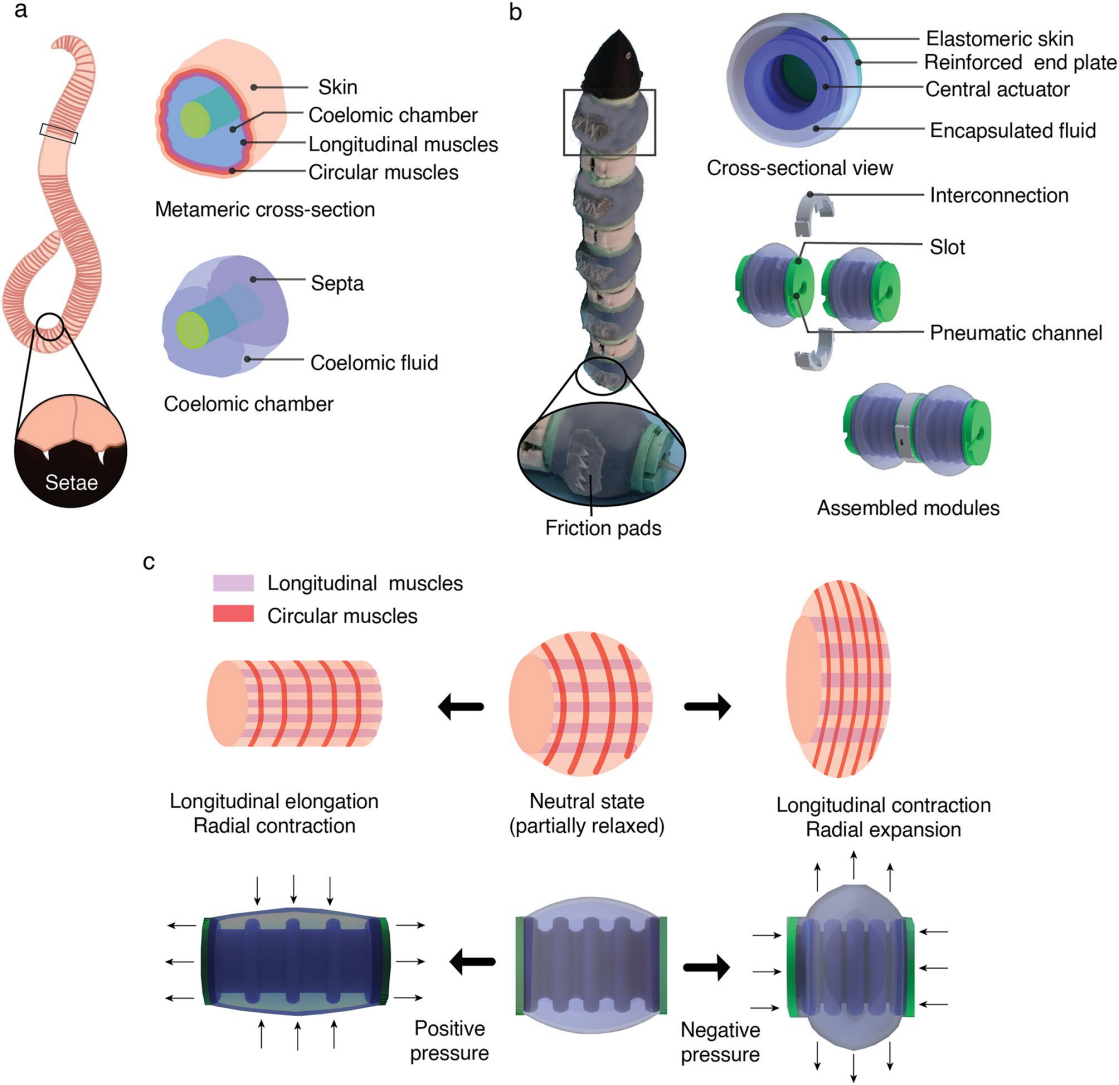
Design translation of bioinspired principles to a soft actuator module and a crawling soft robot. (**a)** Anatomy of earthworm segment cross-section showing constant volume coelomic chambers and setae. (**b)** Bioinspired PSA modules assembled in series using interconnections to form a soft robot with passive setae-like friction pads on its ventral side. **(c)** Working principle of the actuator with positive and negative pressure compared to the muscular motion observed in earthworm segments.

Identifying the attributes of earthworm anatomy that are responsible for subsurface locomotion is important when translating the design principles in the development of the bio-inspired soft robot. As Fig. 1a shows, the muscle-reinforced membrane (septa) runs transversely and separates the individual segments. This is the main method of providing independent motion in the modular design of the actuator. Figure 1b shows that the modular design consists of a central bellows-like actuator that can elongate and compress. The bellow in the middle and the elastomeric skin encapsulates a known amount of fluid, thus mimicking the constant volume of internal coelomic fluid in earthworms. As illustrated in Fig. 1c, the earthworm segment becomes shorter longitudinally and wider circumferentially and exerts radial forces as the longitudinal muscles of an individual constant volume chamber contract. Antagonistically, the segment becomes longer along the anterior–posterior axis and thinner circumferentially with the contraction of circumferential muscles, resulting in penetration forces along the axis.

During the actuation phase, the PSA mimics the muscle movement of the earthworm, as shown in Fig. 1c. The central actuator elongates with positive pressure, causing the elastomeric skin to stretch longitudinally and produce radial compression by pushing the encapsulated fluid inward. Once the pressure has been released, the actuator returns to its neutral position and maintains the shape predefined by the elastomeric skin. To achieve full radial expansion, negative pressure is applied, which compresses the actuator along the longitudinal axis and pushes the fluid radially outward. This PSA configuration change is shown in Supplementary Video S1.

Our design approach and actuation principle represent a new type of soft actuator that can be independently controlled and adjusted to demonstrate earthworm-like bidirectional peristaltic motion. Not just bidirectionality, but the change in forces due to the presence of the bio-inspired constant volume fluid in the actuator makes the design novel. ​Passive friction pads simulate the earthworm's setae and are attached to the ventral side of each PSA module at the maximum diameter to create anisotropic friction during locomotion. The robot created by assembling individual PSAs clearly demonstrates how biology can be translated into a soft robotic system.

### Experimental characterization and performance of PSA

**Figure 2:**
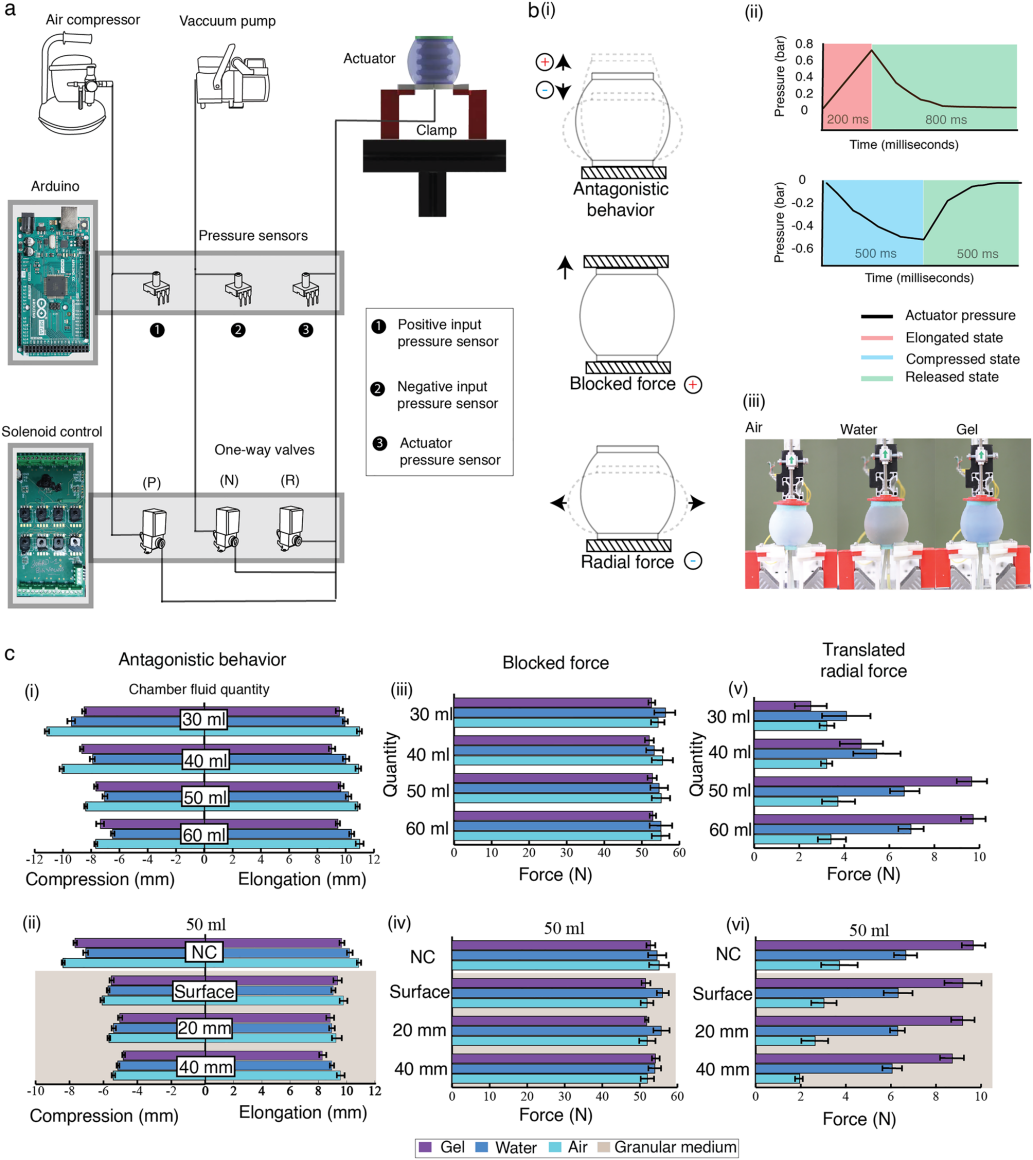
Characterization of peristaltic soft actuator (PSA). (**a)** Schematic of pneumatic system for characterization experiments, One-way Valves: positive pressure (P), negative pressure (N) and release (R). (**b)** (i) Simplified illustration of the characterization type (antagonistic behavior, blocked force, radial force). (ii) Actuator pressure variation with time for positive and negative pressure (iii) Variation in internal fluid for characterization (air, water, gel). (**c)** Characterization results for modules with varying internal quantity and type of fluid in two different conditions NC (Normal Condition) and granular media (i) The air-filled module recorded higher deformation (both in elongation and compression) than both gel and water due to higher compressibility and a lower bulk modulus. An increase in the volume of fluid resulted in fewer deformations; (ii) a steady decrease in the deformation of the actuator with an increase in depth; (iii) blocked force recordings showed no variation with the change in fluid type and volume; (iv) the depth increase in the blocked force did not vary; (v) the increase in fluid quantity resulted in an increased force value for water and gel-filled modules but not for air-filled modules; (vi) all modules recorded lower forces with an increase in depth.

We evaluated the antagonistic behavior (elongation and compression) and force characteristics (blocked and radial forces) under antagonistic motion of the actuator modules. In the experiments, we varied: (1) the type and quantity of the encapsulated fluid in the elastomeric chambers; and (2) the experimental medium. Figure 2a shows the experimental scheme of the characterization, which is explained in Supplementary Fig. S1 along with a data visualization of all the experiments. Figure 2b(i) illustrates the motion of the peristaltic soft actuator during each of the characterization experiments. An Aurora electromagnetic tracer system (Northern Digital) as shown in Fig. 5a(i) was used to accurately track the antagonistic deformation of PSA. A custom setup was used to measure the blocked force and radial force, as described in the Methods section and illustrated in Fig. 5a(ii, iii). For the experiments in granular medium, the granules were filled to a horizontal level with a fixed marker on the side of the box signifying the depth (20 mm or 40 mm) from the top surface of the module. The actuator internal pressure variation for a single cycle is shown in Fig. 2b(ii). The actuator modules used air, water and carbopol gel as the internal fluids, as shown in Fig. 2b(iii).

### Antagonistic behavior (elongation/compression)

Antagonistic behavior is the key principle of peristaltic motion. To investigate this principle, we recorded the motion of the actuator by changing the type (air, water, gel) and volume (30 ml, 40 ml, 50 ml, 60 ml) of the encapsulated fluid. Starting from the lowest volume with 30 ml of air, as shown in Fig. 2c(i) (antagonistic behavior), we found a maximum deformation of 11.13 mm in compression and 10.98 mm in extension (PSA resting length = 50 mm). With a subsequent increase of internal air volume from 30 to 60 ml, the deformation decreased steadily from 11.13 to 7.63 mm during compression. As expected, due to the lower compressibility and higher bulk modulus, the water-filled modules recorded a lower elongation of 9.95 mm and a compression of 9.39 mm, while the gel-filled actuator recorded an even lower elongation of 9.55 mm and a compression of 8.5 mm when filled with 30 ml fixed quantity of fluid. Regardless of the fluid, deformation during compression steadily decreased with increasing fluid volume, which was more pronounced for the air- filled modules than for those filled with water or gel. The amount of fluid creates a resistance to motion and therefore significantly reduces the deformation during compression. However, when elongated, the actuator shows no significant change when the amount of fluid is increased, regardless of the type of fluid.

To estimate the operating range, we conducted an experiment in which the pressure was increased from 0.6 to 1.5 bar, and the elongation of a module filled with 50 ml of air varied from 8.03 to 13.66 mm (Supplementary Fig. S2). A similar experiment was performed for compression. The negative pressure was increased from 0.4 to 0.6 bar, and the deformation varied from 9.86 to 13.15 mm (Supplementary Fig. S3).

Figure 2c(ii) illustrates the effects of the compactness of the granular medium through resistance to motion. "Surface" represents the experiments conducted in a granular medium, in which the module is immersed in granules up to its apex. We measured the high radial force values for the actuator filled with 50 ml and 60 ml of fluid, as reported in the next section on force response. The experiments were therefore conducted with the granular medium for these two volumes. Figure 2c(ii) shows the deformation behavior of the actuator filled with 50 ml of fluid in the granular medium. The performance of the 60 ml-filled module in this medium is described in Supplementary Fig. S4. For the module filled with 50 ml of air, the recorded deformation was measured at a compression of 8.39 mm under normal condition and decreased to 6.08 mm when the module was covered with granules. When the same module was immersed in granular medium at depths of 20 mm and 40 mm, the compression deformation decreased to 5.67 mm and then to 5.42 mm. In terms of elongation, the deformation decreased from 10.86 mm under the normal condition to 9.78 mm when the module was completely buried with granules, and the measured deformations were 9.24 mm and 9.57 mm when the depth of the medium increased from 20 to 40 mm, respectively, with a significant standard deviation.

As expected, both compression and elongation measurements for the water and gel-filled modules showed a similar decrease in deformation with increasing depth. The denser fluids exhibited lower degree of deformation in both elongation and compression for all depths. For example, the elongation recorded for air-, water-, and gel-filled modules at a depth of 20 mm were 9.24 mm, 8.94 mm, and 8.84 mm, respectively, and the compression was 5.67 mm, 5.4 mm, and 5.04 mm, respectively.

### Force response of PSA

The blocked force measurements were performed using the setup shown in Fig. 5a(ii) as explained in the Methods section. From Fig. 2c(iii) it can be seen that the blocked force was in the range of 50–60 N when actuated at a constant positive pressure of 1 bar, showing no relationship between the type and volume of internal fluid with the variation of the blocked force. The blocked force is directly related to the size of the central part and thus when the ends of the actuator are securely clamped and the pressure acts on the fixed dimension central actuator, the force values show no variation.. Figure 2c(iv) shows that the force values remained the same for a granular medium with an internal fluid volume of 50 ml and 60 ml, regardless of the type of fluid. The blocked force gives an estimate of the force exerted by an actuator in the longitudinal direction on the subsurface motion, which is critical for loosening the medium before lowering the resistance for forward motion.

Modules filled with internal fluid have a symmetrically curved shape. When vacuum is applied, along with the collapse of the central actuator, the module generates radial force. This radial force measurement can be difficult, due to the unconventional curved shape and softness of the module. We measured the radial force during intermittent actuation with a negative pressure of 0.5 bar using the setup shown in Fig. 5a (iii). This technique is explained in the Methods section. The graph in Fig. 2c(v) shows the load cell values recorded during the experimental measurements. The data indicate that the amount and type of fluid has a significant effect on the generation of the radial force. For a fixed volume of 30 ml, all liquids exhibit a force in the 2–4 N range. The air-filled modules were expected to exhibit the lowest force, as air is highly compressible, but as Fig. 2c(v) shows the gel-filled modulus exhibited the lowest force of the three. This anomaly is due to the gel being highly viscous, and 30 ml did not fill the entire internal volume homogeneously, thus producing an unreasonable force value of 2.52 N. The water modules demonstrated the maximum force value of 4.1 N, due to the homogeneous filling. The radial force load cell data showed the effect of the change in volume of internal fluid for the air module, and no change in the force value was observed. The water- and gel-filled modules showed significant increases in force values when the volume of internal fluid was increased, but the values increased considerably for the 50 ml and 60 ml gel-filled modules, at 9.65 N and 9.72 N compared to 5.73 N and 6.2 N for water.

Force experiments with the granular medium were performed based on the volume of the encapsulated liquid producing the maximum radial forces. Figure 2c(vi) shows that the radial force decreased from 9.65 N to 9.2 N for the module filled with 50 ml of gel measured in air and in the module completely filled with the granular medium, respectively, due to the increasing compaction of the external granular medium. When the depth was further increased by adding more granular medium, forces of 9.19 N and 8.73 N were measured for depths of 20 mm and 40 mm, respectively. The water- and air-filled modules showed a similar decrease in force values. Radial force is important for the displacement of the granular medium, for reducing the resistance of the medium to the sides, and for anchoring in constricted channels. The gel clearly showed better results in generating the radial force and was therefore chosen as the medium to fill the modules. Data for modules filled with 60 ml of liquid are given in Supplementary Fig. S4. The modules filled with 50 ml and 60 ml of gel gave force readings of 9.2 N and 9.5 N, respectively for the experiments with the granular medium when the module was filled to its top surface with granules. Experiments performed at a depth of 20 mm, the 50-ml module showed a force measurement of 9.19 N, while 9.21 N was observed for the 60-ml module. Based on these similar force measurements and levels of performance, the 50-ml module was selected as the optimal amount, as this reduces the possibility of liquid leakage and reduces the weight of the whole system.

### Locomotion performance and experimental validation

**Figure 3:**
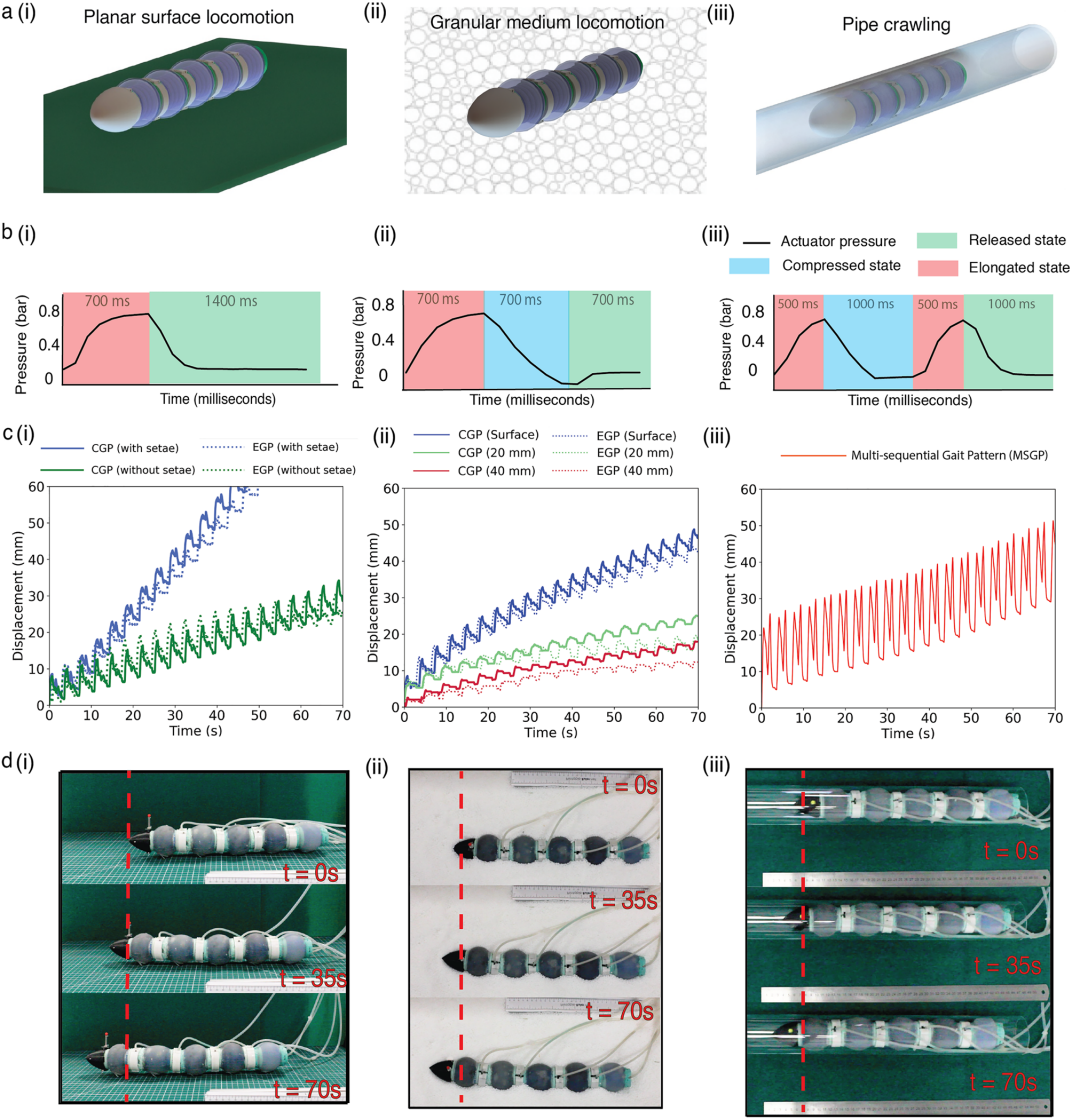
Locomotion performance. (**a)** Pictorial representation of robot movement in different mediums (i) planar surface (ii) granular medium (iii) inside a pipe. (**b)** Actuator pressure variation with time with different gait patterns in the first module of robot for a single cycle (i) elongation gait pattern (EGP) (ii) combined gait pattern (CGP) (iii) multi-sequential gait pattern (MSGP). (**c**) (i) Locomotion performance illustrating the effect of setae and comparison of CGP and EGP in planar surface locomotion (ii) Locomotion performance in granular medium showing the comparison of CGP and EGP on the surface and at depths of 20 mm and 40 mm. (iii) Locomotion performance of the robot in a pipe. (**d)** Locomotion in different environment with time stamps (i) planar surface (ii) granular medium surface (iii) pipe crawling.

From the characterization results, we found that the gel-filled actuator module (50 ml) showed the best performance in force characterization. Five identical actuators were thus fabricated and connected in series to form the modules of a soft crawling robot that can move on a planar surface (Fig. 3a(i)), in a granular medium at different depths (Fig. 3a(ii)), and in a pipe (Fig. 3a(iii)). Two different gait patterns, the elongated (EGP) and the combined (CGP), were used in the locomotion experiments on a flat surface and in a granular medium, while a completely different multi-sequential gait pattern (MSGP) was used for crawling in the pipe. The change in the configuration of the robot for different gait patterns is explained in Supplementary Fig. S5. The pressure changes during one cycle of locomotory wave propagation in the first actuator of the robot for EGP, CGP and MSGP have been shown in Fig. 3b(i), (ii) and (iii) respectively.

The robot was moved using both EGP and CGP on the planar surface. The soft surface of the robot body was not able to generate sufficient friction, with a minimum velocity of 0.45 mm/s and 0.5 mm/s recorded for EGP and CGP, respectively. To increase the anisotropic friction and improve the locomotion performance, passive friction pads like the setae of the earthworm were attached to the ventral side of the actuator modules at the maximum diameter. Figure 3c(i) shows that EGP and CGP exhibited velocities of 1.25 mm/s and 1.35 mm/s, respectively. The effects of the setae and gait patterns on the change in locomotion velocity can be seen in Supplementary Video S2. Initial locomotion experiments were done with the setae placed below the interconnections rather than below the modules and Supplementary Fig. S6 illustrates the difference in performance based on the position of the setae.

During locomotion on granular medium, the loose granules on the surface act like a fluid and provide a very different interaction with the medium than a flat surface. As Supplementary Fig. S7 and Supplementary Video S3 show, the friction pads had no significant effect on increasing the speed of the robot on the surface of a granular medium thus experiments at different depths were performed without the setae. Figure 3c(ii) shows that the CGP achieved higher velocities at different depths (20 mm and 40 mm), as on the flat surface. On the surface, the velocities of EGP and CGP were almost the same (0.68 mm/s and 0.71 mm/s), but within the granular medium, CGP performed better. The active radial expansion in CGP helped to displace and loosen the medium radially outward, resulting in faster motion. As Supplementary Video S3 shows, CGP recorded a velocity of 0.36 mm/s compared to 0.28 mm/s for EGP at a depth of 20 mm. At a depth of 40 mm, velocity recordings of 0.26 mm/s and 0.17 mm/s indicated the same level of advantage. As expected, the speed of the robot decreased with increasing depth due to the increase in compaction. We found that dual configuration increases locomotion efficiency and provides additional locomotion gait patterns that can be explored.

When locomoting in a confined environment, such as a 60 mm inner diameter pipe (robot resting diameter = 60 mm), the robot must release, move, and anchor through the pipe walls. The multi-sequential gait pattern (MSGP) with positive and negative pressure allows the robot to move away (stretch longitudinally) and anchor (extend radially). The traction created by the negative pressure can allow the modules to anchor to pipes with even larger diameters. MSGP was effective only within a constrained environment like a pipe whereas it failed in planar surface and granular medium as shown in Supplementary Fig. S8. The robot moved through the tube at an average speed of 0.65 mm/s, as shown in Supplementary Video S4 and the result was plotted in Fig. 3c(iii). Figure 3d(i), (ii), and (iii) demonstrate the robot's locomotion with time stamps over a total time of 70 s in different media. An overall comparison of the locomotion speeds is given in Supplementary Fig. S9. Our analysis of the experimental locomotion data shows that the initial implementation of a dual configuration in our design can be used to develop different locomotion sequences, thus making the robot suitable for locomotion in different environments.

## Discussion

To demonstrate the locomotion capabilities of robots in environments such as the earthworm's natural habitat, a thorough understanding, and application of its locomotion mechanics is essential in the development of a soft robot. In this work, we propose a peristaltic soft actuator that implements the antagonistic muscle movements of the earthworm by using a single input channel to perform both elongation and compression. The PSA demonstrated a maximum elongation of 10.97 mm at 1 bar of pressure and a maximum compression of 11.13 mm at 0.5 bar of pressure. Table 1 shows some of the recent publications related to pneumatically powered soft peristaltic robots^20,32,33,47,49^ with antagonistic motions in a single module, and to robots^45,46,48,50,57,58^ that demonstrate similar motion using two modules. Thus, most soft modules on pressurization show active deformation in one direction, For example, the actuators used in Zhijie Tang et al.^47^ provide an elongation of 6.7 mm on actuation and those in Yuki Tanise et al^33^ achieve a compression of 15 mm, unlike the bidirectional soft actuator we present in this work.

**Table 1:** Pneumatically powered soft peristaltic robots and their performance.

Authors	Elongation (%)	Compression (%)	Force response (N)	Identical/non-identical modules	Locomotion speed (mm/s)
This work (PSA)	21.9	22.3	E_f_—56.3 C_f_—9.72	Identical	P_l_—1.23 G_r_—0.71 P_i_—0.65
R. Das et al. ^20^	8.3	8.6	N/A	Identical	P_l_—12.42 ± 3.7 G_r_—4.38 ± 0.8
Zhijie Tang et al. ^47^	9.1	N/A	N/A	Identical	P_l_—0.67 P_i_—0.5
Joey Z. Ge et al. ^45^	33.6	N/A	N/A	Non-identical	P_l_—7
S.P. Murali Babu et al. ^49^	N/A	N/A	N/A	Identical	P_l_—4.6 P_i_—6.2
Yasemin O. Aydin et al. ^46^	N/A	N/A	N/A	Non-identical	P_l_—11.0 ± 1.6 (mm/cycle)
S. Yamazaki et al. ^57^	N/A	23.6	E_f_—78.7	Non-identical	P_i_—4.38
Xiongbing Zhou et al. ^48^	87.5	N/A	N/A	Non-identical	P_l_—35 (mm/cycle)
Holam Heung et al. ^50^	N/A	N/A	N/A	Non-identical	P_i_—1.34
Yuki Tanise et al. ^33^	N/A	28.3	N/A	Identical	P_i_—5.84
Ariel A. Calderon et al. ^58^	32.5	N/A	N/A	Non-identical	P_i_—5
Megumi Ikeuchi et al. ^32^	N/A	11	N/A	Identical	P_i_—8.9

*E* elongation, *C* compression, *P*_*l*_ planar surface, *G*_*r*_ granular surface, *P*_*i*_ pipe crawling, *E*_*f*_ longitudinal force, *C*_*f*_ translated radial force.

As explained in the design translation section, a constant volume of fluid is encapsulated in the PSA, as in the coelomic chamber of the earthworm, which can produce longitudinal and radial force when actuated with positive and negative pressure, respectively. This action is analogous to the antagonistic muscular motion on the hydrostatic skeleton of the earthworm, which generates bi-drectional forces^11–14^. In addition to generating force, a constant fluid volume approach also helps us to understand the significance of the fluid in worm locomotion. The PSA recorded a maximum blocked force of 56.3 N when actuated at a 1 bar pressure. The PSA module recorded a tensile force on the loadcell of 9.7N due to radial expansion when actuated with a negative pressure of 0.5 bar. The peristaltic actuator applied in the robot of S. Yamazaki et al.^57^ achieved a blocked force of 78.7 N. In this case the initial configuration was of a radially extended state, so this phase acted passively during release and did not generate radial force. Thus, unlike other actuator modules, the PSA can generate both a longitudinal and a radial force in a single actuator module.

After a full characterization of the PSA, the optimal volume was deemed to be 50 ml and gel was selected as the encapsulated fluid for the actuator, due to its force generation effect. Five similar modules were developed and connected using links to form the soft peristaltic robot. Our initial robot locomotion experiments showed that the friction generated by the elastomer surface with the interacting medium was insufficient to propel the robot. A similar phenomenon occurs in nature, as the streamlined, slippery body of the earthworm is unable to generate enough friction for locomotion, so small bristle-like hairs are used^18^. Thus, inspired by the setae of earthworms, small passive friction pads were attached to the ventral surface of the robot and their effect on locomotion performance was analyzed. S.P. Murali Babu et al.^49^ demonstrated that the speed of crawling locomotion is determined by the shape of the bristles. Bristle-like projections similar to earthworm setae have been used in other robots^46,48^ to generate friction. Our robot demonstrated improved locomotion, with a speed of 1.35 mm/s with a combined gait pattern using both positive and negative pressure over, 1.25 mm/s shown by the elongation gait pattern using only positive pressure. In this work two sequential gait patterns were chosen to compare the effect of the dual configuration during locomotion.

In one of the seminal works^66,67^ on gait analysis, a kinematic model was developed and the maximum achievable average speed corresponding to an optimal gait was calculated. In it, it is postulated that gait patterns with a higher limit ratio (ratio of the number of relaxing segments to the number of anchoring segments) have a higher locomotion speed, which was verified by locomotion experiments with a segmented electro-mechanical robot. Our pneumatic soft robot showed similarities in the gait patterns used compared to the previous study. The combined gait pattern (CGP) has an anchoring segment and a relaxing segment at each time interval, as the wave passed through the robot´s body. CGP showed a higher velocity of locomotion compared to the elongation gait pattern (EGP), which is completely consistent with the postulation of a higher limit ratio mentioned in the gait modelling study^66,67^. The studies also found that anchor slippage is an important factor, in determining the performance of the robot.

In the future more in-depth study would be done exploring different gait patterns to find the optimum gait pattern that reduces slip^37^ and shows better velocities. The change in size^71^, shape^72^ and quantity^73,74^ of granules have a direct relation to the compaction experienced by an intruder traversing in a granular medium. In biological literature, we have seen organisms based on their size and external compaction, change their burrowing strategies^75,76^. To avoid the high cohesive forces in natural soil and ease the repeatability and handleability of experimentation, similar to other works^25,26^, we chose a commercially available granular bead as a medium for testing locomotion. Granular surface experiments indicated that unlike on a planar surface, the friction pads had no significant effect in enhancing the locomotion performance within a granular medium. The robot was able to move on the granular surface and within the medium at depths of 20 mm and 40 mm, achieving maximum speeds of 0.71 mm/s, 0.36 mm/s, and 0.26 mm/s respectively with the combined gait pattern. Several robots^20,45,49^ have demonstrated better velocities on planar surfaces, but the bioinspired soft robot design of our proposed system represents a significant development, as it enables the robot to travel within a granular medium. When actuated with CGP, the robot can perform repeated penetration expansion cycles, equivalent to the earthworm’s locomotion in the soil, by modulating the coelomic fluid-filled chamber^17^. Our previous robot demonstrated a better locomotion velocity at shallow depths of 20 mm, but for depths of 40 mm it was unable to locomote, whereas our new system moved with a velocity of 0.26 mm/s. This also implies that our bioinspired design can prove to be effective for locomoting in deeper and more compact regions. This work focused on movement through granular medium whose physical properties differ from real soil and thus future work will involve moving inside soil and studying the effect of burrowing using crack propagation^15,73^.

The robot was also able to crawl through a tube with a 60 mm inner diameter, demonstrating a velocity of 0.65 mm/s. Due to the constricted environment, the EGP and CGP failed to make the robot move and thus a different gait pattern was devised with multiple segments actuated to make the robot anchor and move forward. The active radial expansion also allows the robot to anchor itself onto pipes of larger diameters. Crawling through pipes has been previously observed in other pneumatic robots such as that of Yuki Tanise et al.^33^ and Megumi Ikeuchi et al.^32^ with better velocities (5.84 mm/s and 8.9 mm/s), but the versatility of the robot, illustrated by its ability to move in different media, is unique in our system. As future work, similar to other peristaltic robots^30,46,77,78^, we will modify the design of individual actuators and gait patterns, enabling our robot to locomote in a 2D space compared to current uniaxial rectilinear locomotion. The modified modules would be able to perform omnidirectional bends, allowing the robot to steer and perform both rectilinear and undulatory locomotion. In compact environments, undulatory locomotion would result in reduced environmental compaction and locomotion in 2D space.

Our robot has several unique features that may be useful in the development of soft earthworm-inspired robots in the future. The adaptive functionality in the underlying biomechanics of annelid locomotion inspired us in the development of this soft robot. Antagonistic force generation through actuation with positive and negative pressure in a constant volume chamber is unique to our system. By considering a variable configuration, the robot can explore different gaits and be used for locomotion in other confined environments. The gait patterns need to be optimized to understand the effect on the concerned parameters like anchor slippage, locomotion velocity etc. As future work, similar to a previous study^66^, we will create and extend a simple kinematic model in order to develop a standard gait pattern generation function based on the individual operation of the actuator modules. Every segment of the robot needs to be sensorized with a feedback control loop for providing proprioceptive data. The prototype would be actuated, and the data collected would help us in the optimization of gait patterns and validation with the results from the gait generation model.

## Methods

### Actuator design and fabrication

**Figure 4:**
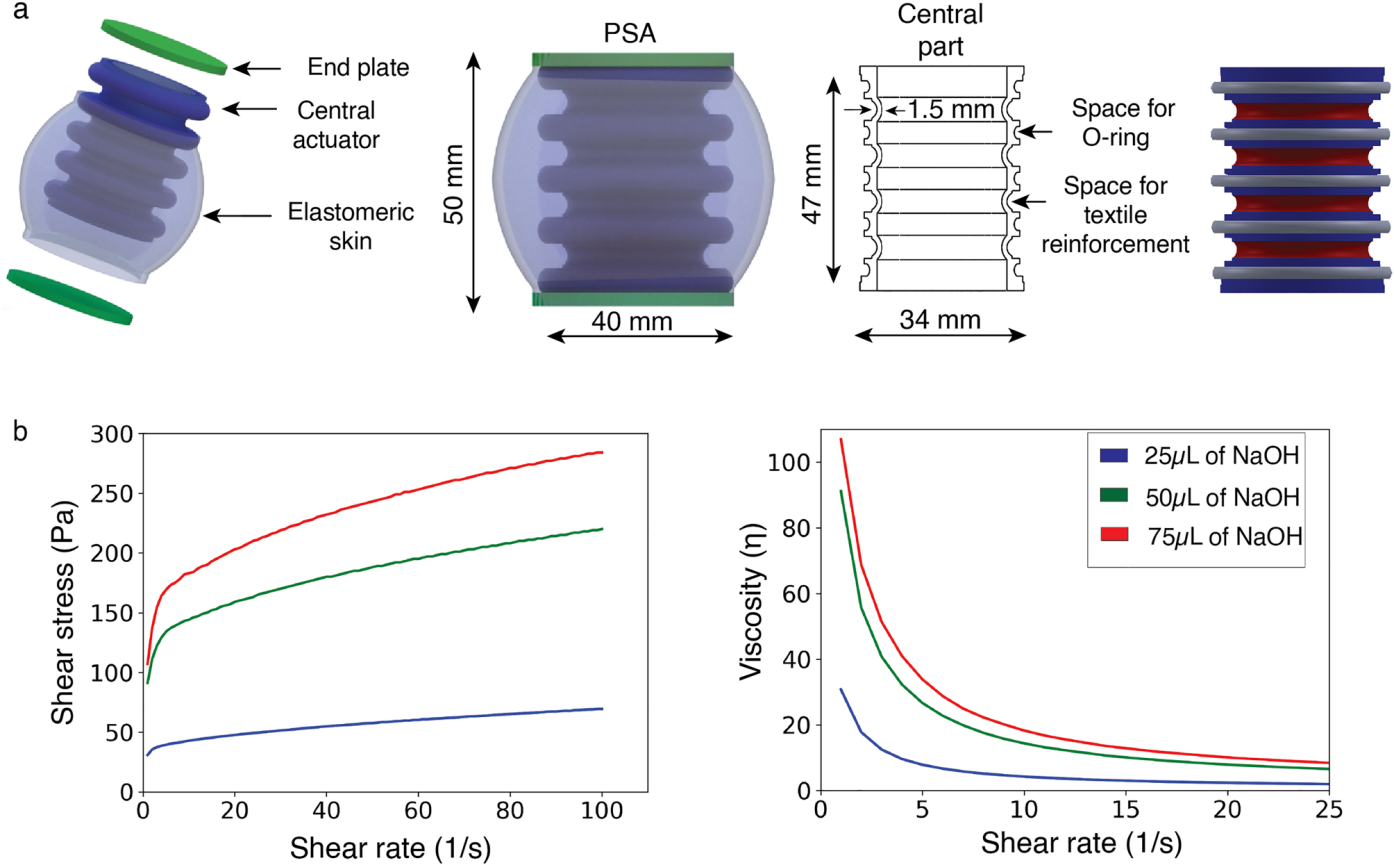
PSA module design and Carbopol gel rheology plots. (**a)** PSA design comprises a bellow-like functioning active central actuator and a passive elastomeric skin, presence of O-ring ensures uniform longitudinal elongation and textile reinforcement prevents bulging at intermediate thin regions. (**b)** Increase in NaOH concentration resulted in higher viscosity and shear stress of the gel due to formation of more polymeric chains and 50 µl was chosen as optimum concentration and used for characterization of the modules.

Antagonistic configuration change was a key consideration in the development of PSA. Bellows are commonly used in industrial applications and soft robotics for longitudinal motions (elongation and compression). In actuation terms, the PSA consists of two parts (Fig. 4a): the central bellows-like actuator, which causes the antagonistic behavior, is the active part; and the external skin, which encapsulates various fluids, acts as the passive part. The central bellows-like actuator consists of four components: the central part, the O-rings, the textile reinforcement, and the external silicone layer. Using SOLIDWORKS 2017, a PSA module was designed with an outer diameter at the ends of 40 mm and a height of 50 mm. A central part, which functions as a bellow, had an inner hole diameter of 27 mm and a height of 47 mm (see Fig. 4a). To align the longitudinal motion with positive pressure, 3D-printed O-rings made of FlexPRO 98 material were inserted into the cavities to provide rigidity to the actuator. In the design of the center section, 1.5 mm-thick curvatures were added to enable it to contract under negative pressure. Textile reinforcement was added along the outer surface of the thin curvatures to prevent bulging during elongation. To prevent the central actuator from collapsing under negative pressure and the O-rings from shifting out of position, an external layer of silicone was added. A 2 mm-thick curved elastomeric skin with an end diameter of 40 mm was designed to provide external coverage of the actuator and encapsulate the fluid, like the earthworm's coelomic chamber. A bulge was designed in the skin to avoid constraints and facilitate compression, displacing the fluid radially and generating force. The end of the skin and the central actuator provided a disc-shaped space with a 2 mm thickness, into which a tube was inserted to access and fill the inner chamber. Reinforcement prevented concave and convex bulges at the circular ends when positive and negative pressures were applied. Details steps for fabricating a single PSA module are given in Supplementary Video S5 and explained in Supplementary Fig. S10.

### Gel preparation

A carbopol-based gel was chosen, which has similar properties to commercial hair gel. For the preparation, 0.8 g of Carbopol powder was mixed with 100 ml of deionized water. The mixture was stirred at room temperature for thirty minutes, and NaOH was added as a thickening agent. To evaluate the properties and identify the ideal viscosity, three samples were prepared with different concentrations of NaOH (25 µl, 50 µl and 75 µl). Rheological analysis of the samples resulted in the graph shown in Fig. 4b. Increasing the concentration of the thickener was found to facilitate the formation of linkages in the polymer chains, leading to an increase in viscosity and shear stress. The 75 µl sample was too viscous to fill the modules. The gel with a concentration of 50 µl NaOH was selected because it had the optimal viscosity to facilitate uptake and extraction into the chamber.

### Experimental setup and methodology

As shown in Fig. 2a, a pneumatic system was set up to operate with both positive and negative pressure. Each measurement involved the collection of values over a period of ten cycles. For all experiments, the module was fixed to the floor by a clamping system of rigid aluminum rods and custom 3D-printed parts to prevent the actuator from slipping. A wooden box measuring 50 cm × 100 cm × 40 cm was filled with homogeneous white elliptical beads (diameter of 4 mm) made of polyoxymethylene (POM; Ultraform N2320 003; BASF, Ludwigshafen, Germany) to simulate and perform the experiments with the granular medium.

#### Antagonistic behavior

**Figure 5:**
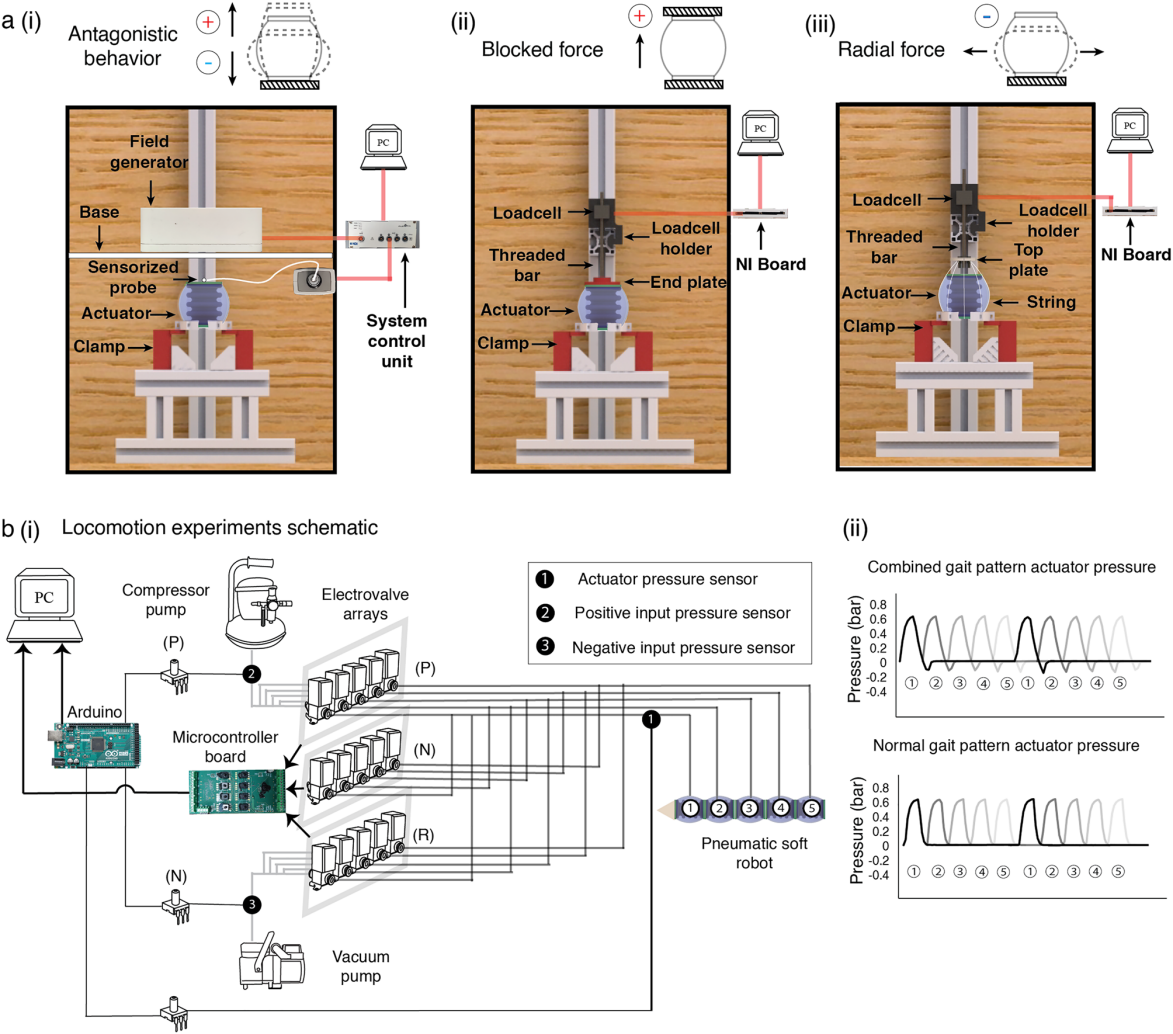
Actuator characterization and locomotion setup. (**a)** (i) Aurora Electromagnetic tracer system (NDI) mounted on top of PSA to measure its deformation when actuated giving an estimate of the antagonistic behavior. (ii) Loadcell with an end plate restricting the longitudinal deformation of the PSA was used to measure its blocked force. (iii) End plate was replaced with a top plate containing 6 equidistant holes. 6 strings attached to the bottom of the actuator passed along the surface of the PSA to the top plate translating the radial force to tensile force for the loadcell. (**b)** (i) Pneumatic setup to perform locomotion experiments containing electrovalve arrays to generate different gait patterns. Schematic (ii) variation of actuator pressure throughout the robot comparing EGP and CGP for two complete locomotion cycles.

To estimate the elongation and compression of the actuator, an Aurora electromagnetic tracer system (Northern Digital) was used as shown in Fig. 5a(i). A low-intensity electromagnetic field is generated by the Aurora Field Generator, which gives a measurement volume. The field generator was mounted on the actuator at a vertical safe height of 20 mm, and a sensor-controlled probe was connected to the top of the actuator. When pressurized by the pneumatic system, the system control unit detects the position and orientation of the sensorized probe within the generated field and records the range of deformation of the actuator. Both the field generator and the sensor-controlled probe were connected to the system control unit, which was operated and monitored via PC. For the measurements in the granular medium, the experimental protocols remained the same.

#### Blocked force

The longitudinal force along the direction of the robot is responsible for loosening the medium while the robot moves forward. As shown in Fig. 5a(ii), a load cell was mounted on the clamped actuator with a threaded rod connected to an end plate. A National Instruments card was connected to the loadcell to monitor the readings in the computer. To measure the blocked force of an actuator, its movement in the longitudinal direction must be completely restricted. When experimenting within granular media, it was necessary to ensure that granules were not trapped between the top of the PSA and the bottom of the end plate.

#### Radial force

As shown in Fig. 5a(iii), an innovative technique was used in which the threaded rod, like the blocked force, was connected to a top plate with six holes fixed at a height of 15 mm from the head of the module. Six strings attached to the bottom of the module ran longitudinally upward along the skin. The strings were passed through the six 2.6-mm-diameter holes running concentric to the top plate (Supplementary Fig. S9). When negative pressure is applied, the deformation is recorded in the load cell data and the force measurement can be estimated from the data given in Supplementary Fig. S11. During characterization, photographs of the string configuration were taken both before and after actuation. The range of angular deflection, θ, was found to vary about 5° during the compressed phase and the neutral phase of the actuator, corresponding to a variation of 0.12 in the value of the tangent of the angle. Due to the negligible variation, the angular component was neglected for further calculations of the radial force, so that the radial force correlates directly only with the value of the load cell.

#### Locomotion

As shown in Fig. 1b, the PSA modules filled with gel were extended by 5 mm and slotted by 2 mm to allow the lightweight laser-cut connectors to be properly attached to mount five actuator modules in series (robot length = 350 mm). A pneumatic system (Fig. 5b(i)) with three arrays of five electrovalves was set up to perform the motion experiments and generate different gait patterns, as explained in Supplementary Fig. S5. Since the characterization of the PSA was performed at a pressure of 1 bar for elongation and 0.5 bar for compression of each module, the locomotion experiments were performed at an optimal pressure of 0.7 bar for elongation and at the same vacuum pressure of 0.5 bar to avoid damage and leakage of the modules. As shown in Fig. 5b(i), the electrovalve arrays were controlled by the microcontroller board to generate different gait patterns and move the robot in relation to the particular experiment being performed.

For the experiments on flat surfaces, the robot was made to travel a fixed distance of 10 cm while varying two parameters: (i) the gait pattern (EGP and CGP), and ii) the surface friction (with setae and without setae). For locomotion in granular medium, the surface of the granules was first aligned horizontally, and the robot was put into location. A box (50 cm × 100 cm × 40 cm) filled with white granules served as the experimental setup in accordance with the desired height (20 mm or 40 mm), so that the marker was just above the surface of the medium and could be tracked. Visible markers were placed at 20 mm and 40 mm heights on the head of the robot to track its movement when submerged. Videos were analyzed using open-source Tracker software. The pipe crawling experiments were performed in a polycarbonate tube with an inner diameter of 60 mm. Figure 5b shows the variation in actuator pressure as a function of gait pattern through all modules of the robot for a complete cycle. For the pipe crawling experiments, a completely different multi-sequential gait pattern was used (Supplementary Fig. S5).

## Supplementary Information

Supplementary Information.

Supplementary Video 1.

Supplementary Video 2.

Supplementary Video 3.

Supplementary Video 4.

Supplementary Video 5.

## Data Availability

The datasets generated and/or analysed during the current study are available in the “Data Repository: SREP-22-00552A” repository, Weblink: https://osf.io/7xfuv/?view_only=923bc1d4272f4aaba4ca4206af103bc6.
