# Supplementary material for: An earthworm-like modular soft robot for locomotion in multi-terrain environments
Source: Sci Rep. 2023 Jan 28;13:1571. doi: 10.1038/s41598-023-28873-w (PMC9884293; doi:10.1038/s41598-023-28873-w)
Supplement: Supplementary file 1 — Supplementary Information. [file 41598_2023_28873_MOESM1_ESM.docx]

Supplementary Information: An Earthworm-like Modular Soft Robot for Locomotion in Multi-Terrain Environments

Riddhi Das^1,2,*⸸^, Saravana Prashanth Murali Babu^1,3,*⸸^, Francesco Visentin^1,4^, Stefano Palagi^1,2^, Barbara Mazzolai^1,*^

^1^Bioinspired Soft Robotics Lab, Istituto Italiano di Tecnologia, Genova, Italy

^2^The BioRobotics Institute, Scuola Superiore Sant’Anna, Pontedera, Italy

^3^Center for Soft Robotics, SDU Biorobotics, The Maersk Mc-Kinney Moller Institute, University of Southern Denmark, Denmark

^4^Department of Computer Science, Università degli Studi di Verona, Verona, Italy

^*^[riddhi.das@iit.it](mailto:riddhi.das@iit.it), [spmb@mmmi.sdu.dk](mailto:spmb@mmmi.sdu.dk), [barbara.mazzolai@iit.it](mailto:barbara.mazzolai@iit.it)

^⸸^these authors contributed equally to this work

**Actuator characterization pressure**

As shown in Figure 2a, to control the actuators, we used solenoid valves to sequentially switch between positive and negative pressure, and three pressure sensors to monitor the input pressure of the actuator before and after the solenoid valves. The positive, negative and release valves are marked as P, N and R respectively. The positive pressure for measuring the module's elongation and the blocked force was fixed at 1.0 bar with a 200 ms actuation time (opening of positive pressure valve) and an 800 ms release time (closing of positive pressure valve and opening of release valve). Similarly, for measuring the deformation due to compression and the radial force, the vacuum pressure was fixed at 0.5 bar with a 500 ms actuation time (opening of negative pressure valve) and a 500 ms release time (closing of negative pressure valve and opening of release valve). The actuator internal pressure for ten cycles for both positive and negative pressure experiments can be seen in Supplementary Fig. S1.


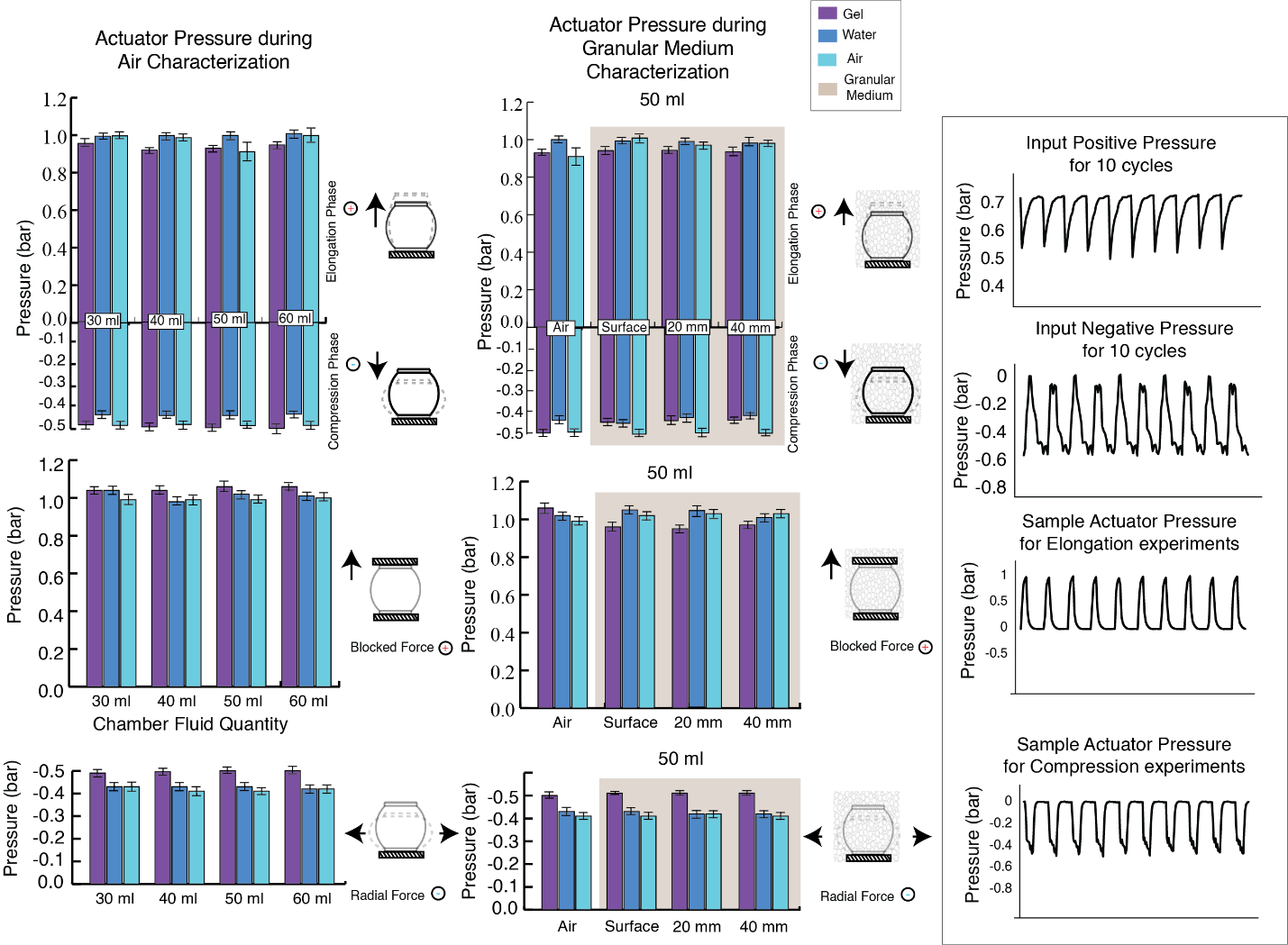


**Supplementary Figure S1**: On the left section the Actuator Pressure variation has been shown for all the experiments performed in air with increase in the volume of encapsulated fluid. The Figure section in the middle demonstrates the pressure variation for experiments done in granular medium. The figures on the right show the pressure variation for ten cycles for Input Positive, Input Negative pressure and Actuator Pressure for elongation and compression experiments

**Ramping experiments to enhance antagonistic behaviour**


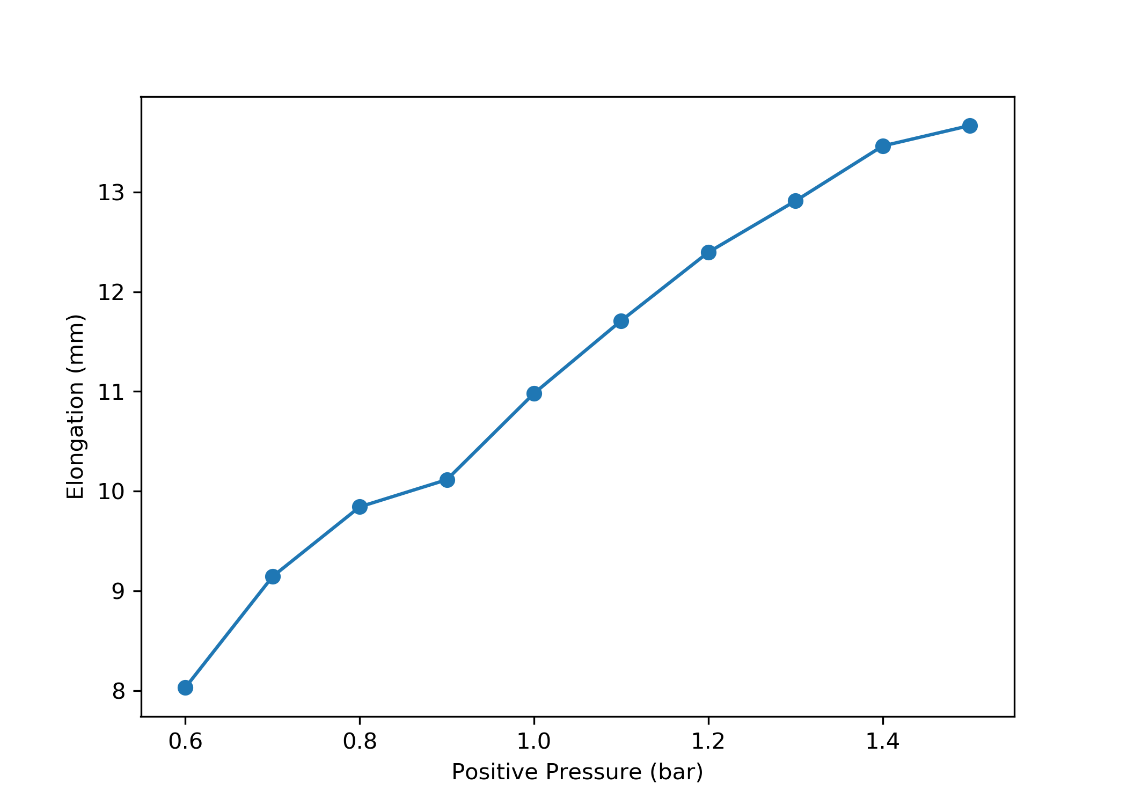


**Supplementary Figure S2:** Ramp Experiments performed measuring the elongation displacement (mm) of the actuator with variation of pressure from 0.6 bar to 1.5 bar has been shown.


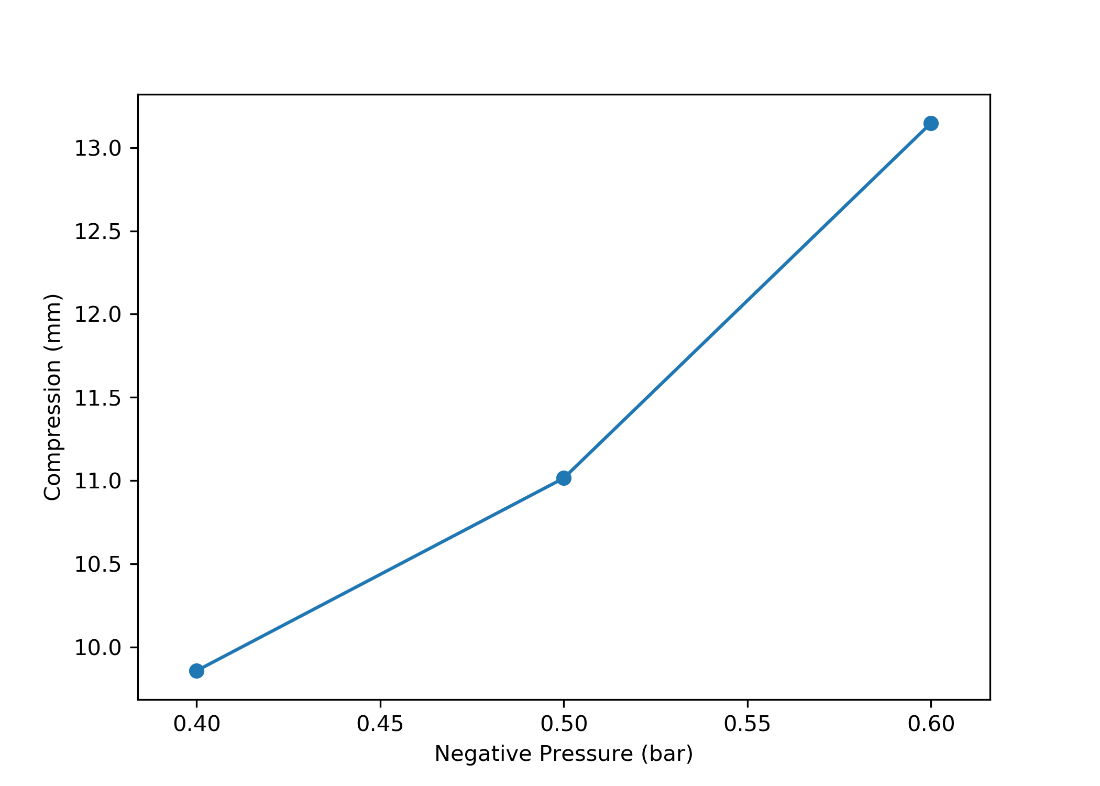


**Supplementary Figure S3:** Ramp Experiments performed measuring the compression displacement (mm) of the actuator with variation of pressure from 0.4 bar to 0.6 bar has been shown.

**Characterization results of 60 ml filled PSA module**


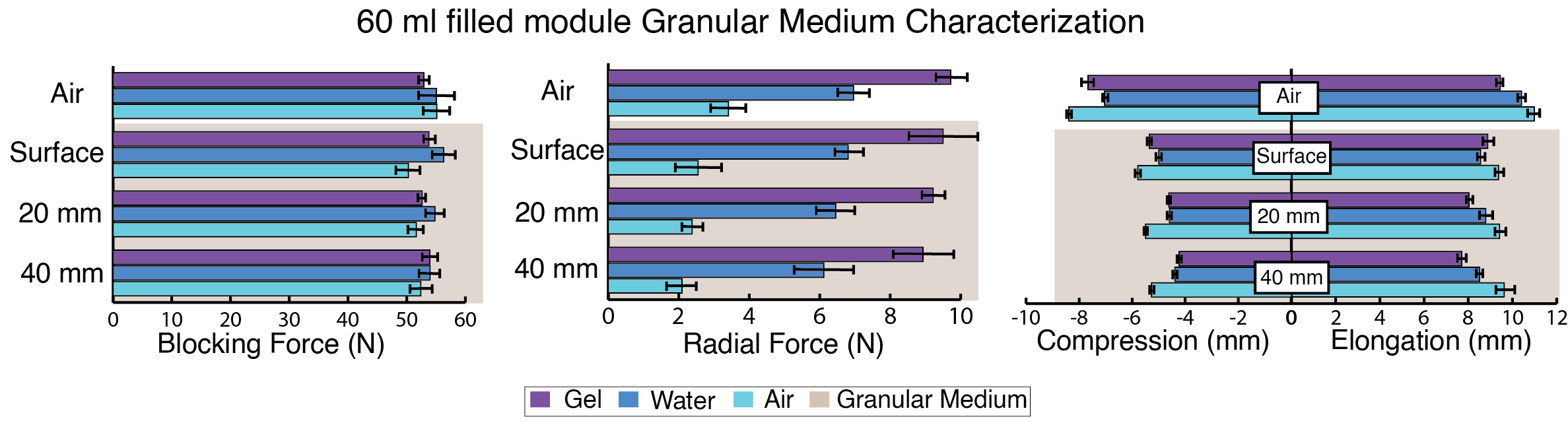


**Supplementary Figure S4:** Results of characterization experiments of the 60 ml filled module in granular medium showed similar characteristics with the 50 ml filled module

**Gait pattern sequences used for locomotion experiments.**

For the locomotion experiments, two gait patterns, elongation (EGP) and combined (CGP) have been used to study the robot's performance in planar surface and granular medium. The robot was also made to locomote in a pipe using a different sequential waveform. The gait patterns are generated by opening and closing the electrovalve arrays in different orders using the Solenoid control board. The positive, negative and release valve arrays are marked by P, N and R respectively. The board was controlled by a software where actuation sequences can be run by simple valve ON/OFF scripts mentioning the delay of every step. From Supplementary Fig. S5, the gait patterns can be understood step by step, visualizing the condition of the robotic prototype in every step. As denoted in the legend, a red bump signifies the activation of a positive pressure valve. The actuated valve number can be realized from the position signified on the robot prototype on top. Likewise, green and blue bumps signify the opening of release and negative pressure valves. The significant difference between EGP and CGP is the use of the negative pressure. The two locomotion gait patterns were written to clearly understand the importance of the implemented dual configuration in the modules. The positive and negative pressure values were kept constant at 0.7 bar and 0.5 bar respectively. As shown in Supplementary Fig. S5, EGP waveform is similar to traditional peristaltic robotic configurations where the modules are actuated one after the other with positive pressure. In the combined waveform, the modules elongating also contracted due to negative pressure before going to the release phase. The EGP actuation time for every module was kept 700 ms, and the release time was fixed to a 1400 ms. The combined waveform for a module starts with an initial actuation of 700 ms by positive pressure, then compression of 700 ms by negative pressure and then release for 700 ms. To have a direct comparison among both the gait patterns the time of actuation for every step was kept 700 ms. In Supplementary Fig. S5, similarly after the red and green lines, the blue line signifies which module is subjected to negative pressure along with the robot's configuration at that exact moment. Both the waveform sequences were tested on a planar surface and in a granular medium. In the case of a granular medium, tests were performed on the surface, within 20 mm depth and 40 mm depth. The robot was also made to move through a pipe of diameter 60 mm. In order to move through a confined space like a pipe the robot needed to perform sequential anchoring with rapid release of the body segments. As shown in Supplementary Fig. S5 an entirely different multi-sequence gait pattern was used to locomote through a pipe where all the first module was actuated with positive pressure for 500 ms, followed by negative pressure for 500ms, positive pressure for 500ms, negative pressure for 500ms, and the valve remained closed for 500ms, followed by positive pressure of 500ms and finally with a release period of 500 ms. The rest of the modules followed different actuation sequences that can be seen from Fig S5. Like the previous gait patterns, the value of the positive and negative pressure was 0.7 bar and 0.5 bar respectively.


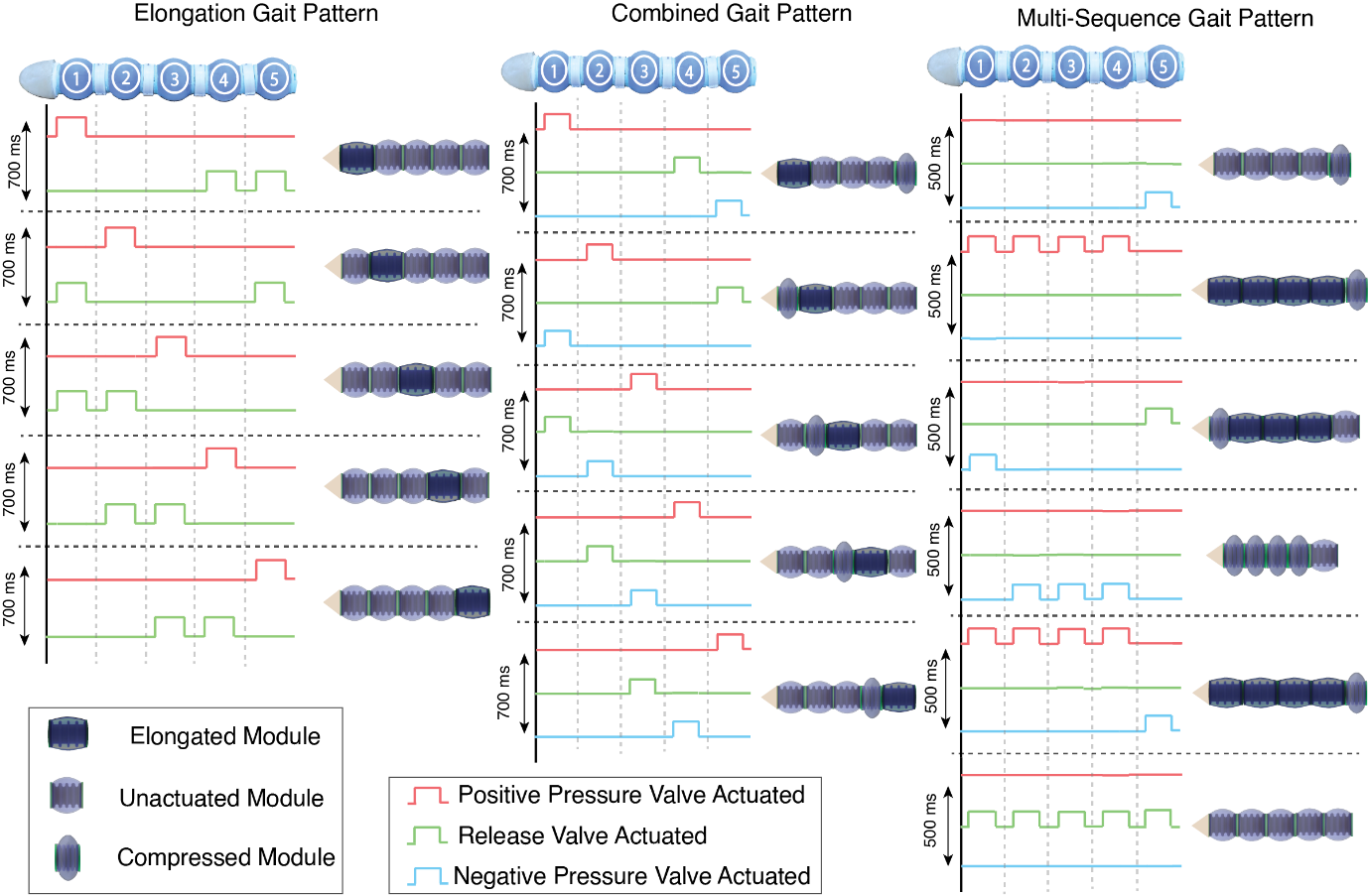


**Supplementary Figure S5:** The figure illustrates the opening and closing of valves during a complete cycle of the locomotion for elongation, combined and multi-sequence gait pattern. The configuration of the robot illustrating the module configurations for an entire cycle has also been shown.

**Effect of change in setae position on locomotion performance of robot on planar surface**


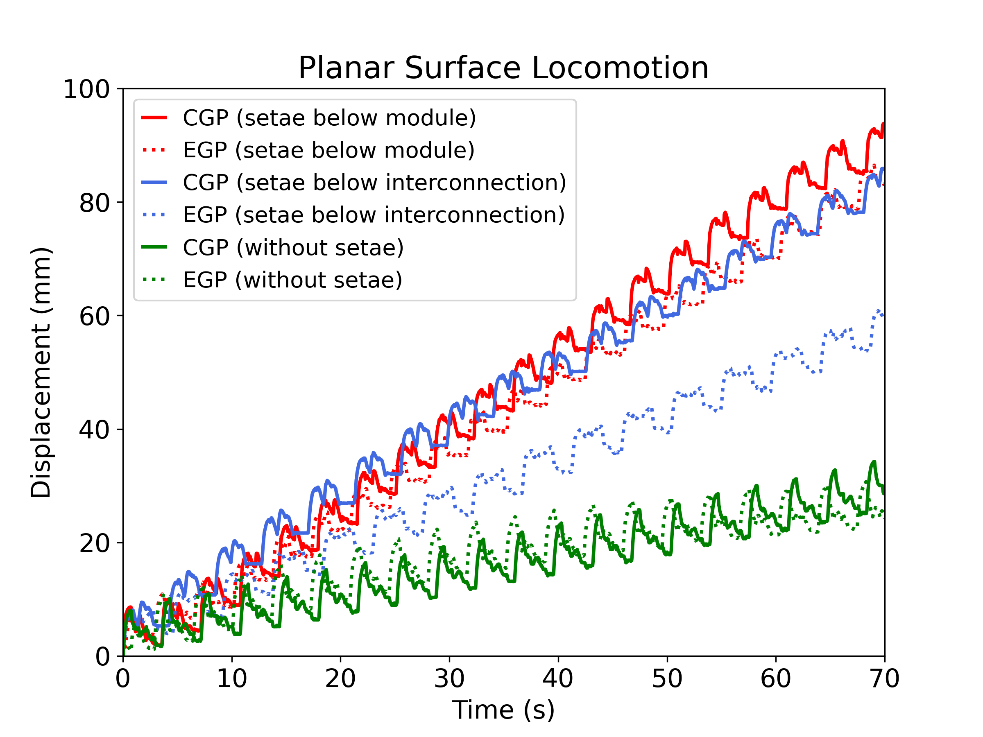


**Supplementary Figure S6:** Change of setae position from bottom of interconnections to the bottom of the modules resulted in enhanced locomotion on planar surface. Setae below module (CGP – 1.35 mm/s, EGP – 1.25 mm/s), Setae below interconnection (CGP - 1.23 mm/s, EGP - 0.88 mm/s), No Setae (CGP – 0.5 mm/s, EGP – 0.45 mm/s)

**Effect of change in setae position on locomotion performance of robot on granular medium**


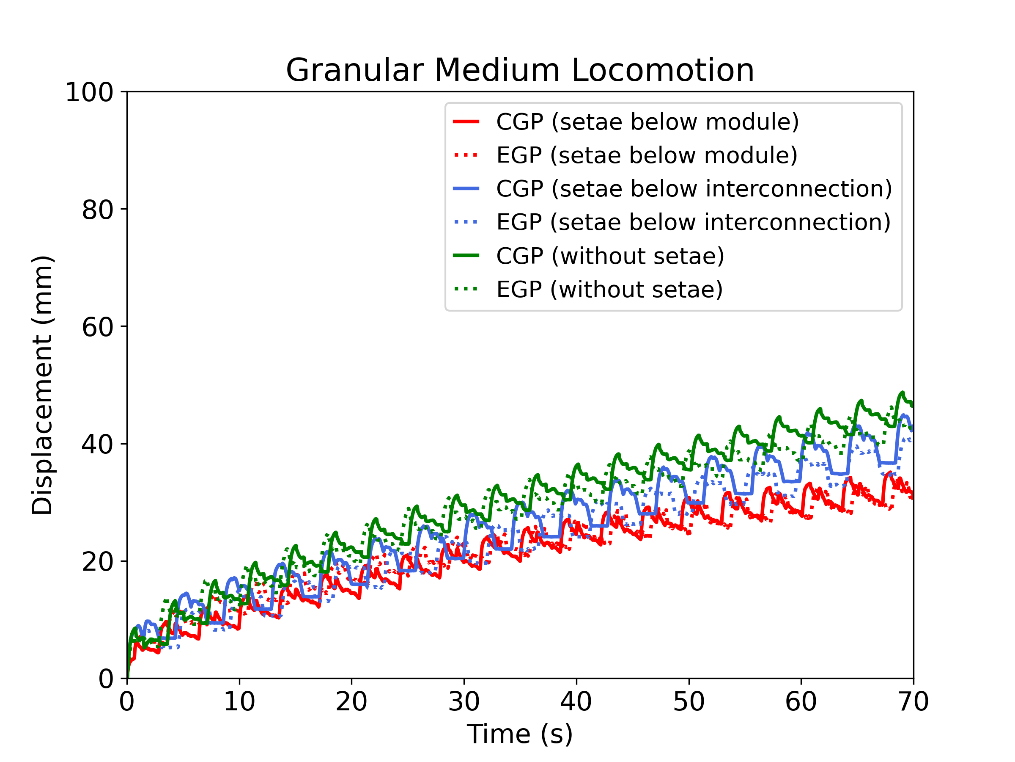


**Supplementary Figure S7:** Change of setae position from bottom of interconnections to the bottom of the modules resulted in diminished locomotion performance on the granular medium surface. Setae below module (CGP – 0.5 mm/s, EGP – 0.49 mm/s), Setae below interconnection (CGP - 0.65 mm/s, EGP - 0.59 mm/s), No Setae (CGP – 0.71 mm/s, EGP – 0.68 mm/s)

**Locomotion performance of Multi-sequence Gait Pattern on planar surface and granular medium surface**


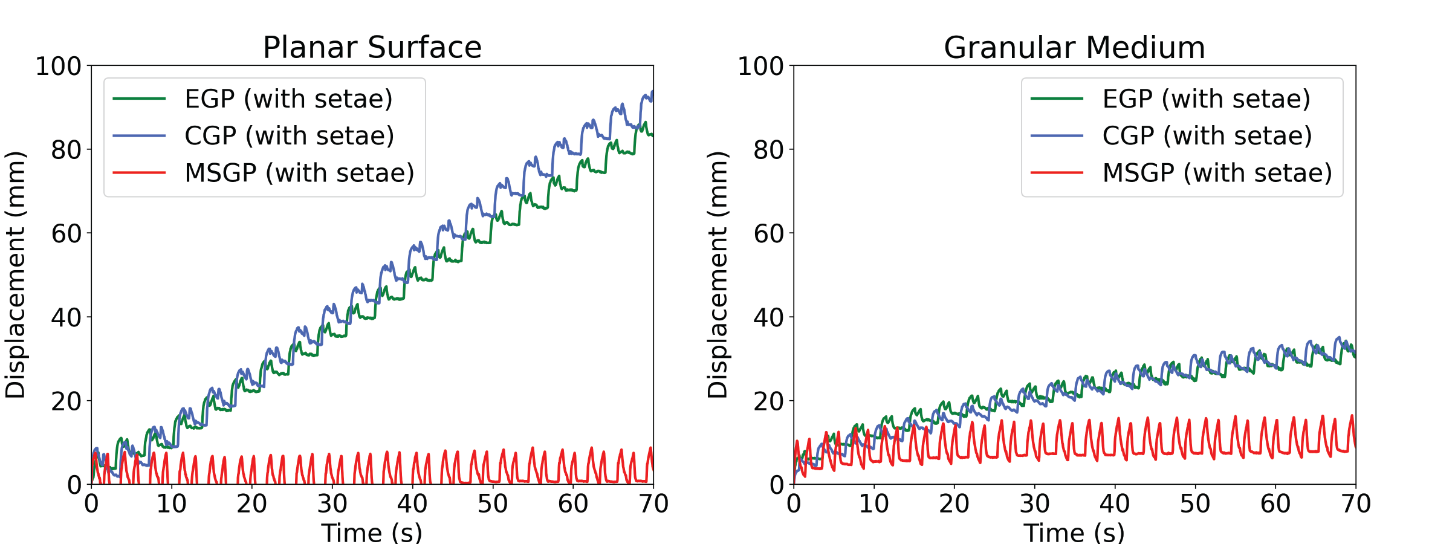


**Supplementary Figure S8:** Multi-sequential Gait Pattern did not work in both planar surface and granular medium.

**Comparison of locomotion velocities in different mediums**


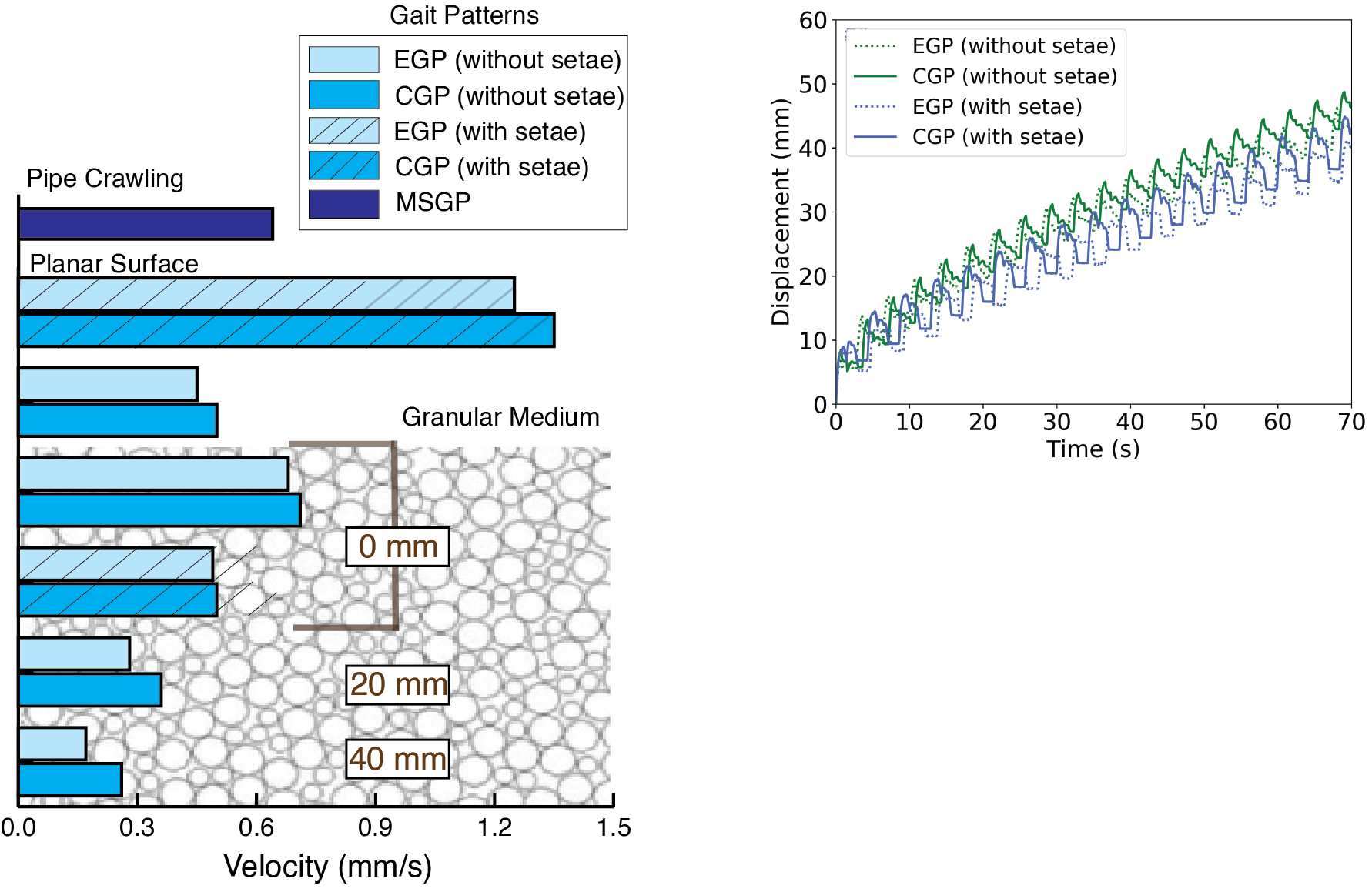


**Supplementary Figure S9:** Comparison of the average locomotion velocities for all the experiments performed in different terrains

**Fabrication steps of a PSA**

The central part responsible for the dual-action is cast in a 3D printed precise mould using DragonSkin-30 (Smooth-ON). Initially, a layer of Mold Release was spray coated on the mould, Dragon Skin 30 was prepared, and a fluorescent pigment of SilcPig (Smooth-ON) was added. After degassing, Step 1 involved the filling of the bottom assembled mould and then the top assembled mould subsequently. During elastomer filling, the mould edges were sealed with the hot glue gun to prevent leakages. After curing at 60° C for an hour, the central casted part was removed from the mould using a pressure gun, as shown in Step 2. In the following step 3, textile reinforcements were glued to specific places. A solution of 5% Acetone and 10 gm Silpoxy (SmoothON) was painted on one side of the textile. The textile strips were glued onto the valleys present in the central part, as shown in Figure 4a. In Step 4, O rings made with Flex-Pro 98 material (RS Pro) were attached in the positions shown in Figure 4a using Sil-poxyTM (Smooth-ON). In Step 5, to fabricate the external layer, Dragon-Skin 30 was cast into another bilayer mould with a two-step casting process similar to Step 1. After the curing process for a similar duration and temperature, the finished central actuator was taken out. To ease the retrieval of the stiff central actuator, the central mould design was modified to be broken up into three separate parts while removing, as shown in Step 6. Now with the completion of the central actuator, we move on with the fabrication of the skin. In Step 7, a 3D printed mould was assembled and filled with degassed Dragon-Skin 10 (Smooth-ON) and cured for 45 minutes at a constant temperature of 60° Celsius. In Step 8, the skin is removed from the disassembled mould. With the skin and the Central Actuator fabricated, the final step is to attach them and complete the PSA. SmoothSil -960 (Smooth-ON) was chosen as the material for the circular ends of the module. After pouring SmoothSil -960 up to a level marked in the End Curing mould, it was allowed to partially cure in the heater for about 40 mins as shown in Step 9. The mould was provided with a hole on the bottom side to add a pipe so that after curing, it can actuate the central bellow. Next, a circular PET sheet with a thickness of 0.70 mm with holes was placed on the partially cured silicone, and then SmoothSil -960 was poured in the end curing mould filling it till a demarcation. The skin and the central part as shown in the Figure 4 are subsequently put in Step 10 and left to cure for an hour in the heater at the temperature of 60° Celsius. After curing on one side, the other side was cured in a similar way mentioned in Step 10. This finished the complete fabrication of one PSA is shown in Step 11. A hole is drilled from the bottom and pipe is inserted to access the internal chamber which is the space between the central actuator and the skin as shown in Figure 4a. By means of the pipe, several fluids can be inserted or extracted to the module. In the case of earthworms, the internal encapsulated fluid varies in constituents and concentration across different species; hence an estimate of the performance with the actuator can be done based on the quantity of fluid and the kind of fluid. The range of the fluid quantity to be tested was chosen as per some preliminary experiments with water. With 20 ml of the internal chamber filled, the module was underfilled and not meant for experimentation. In the case of 70 ml, it was found to overfill since deformation and force experiments resulted in minor changes, and the excess quantity of fluid showed leakage problems. Based on the above two observations range of fluid quantity was varied from 30 ml to 60 ml with 10 ml difference. Standard fluids such as air, water, and gel, incompressible at room temperature, were selected to distinguish the modules' performance clearly.


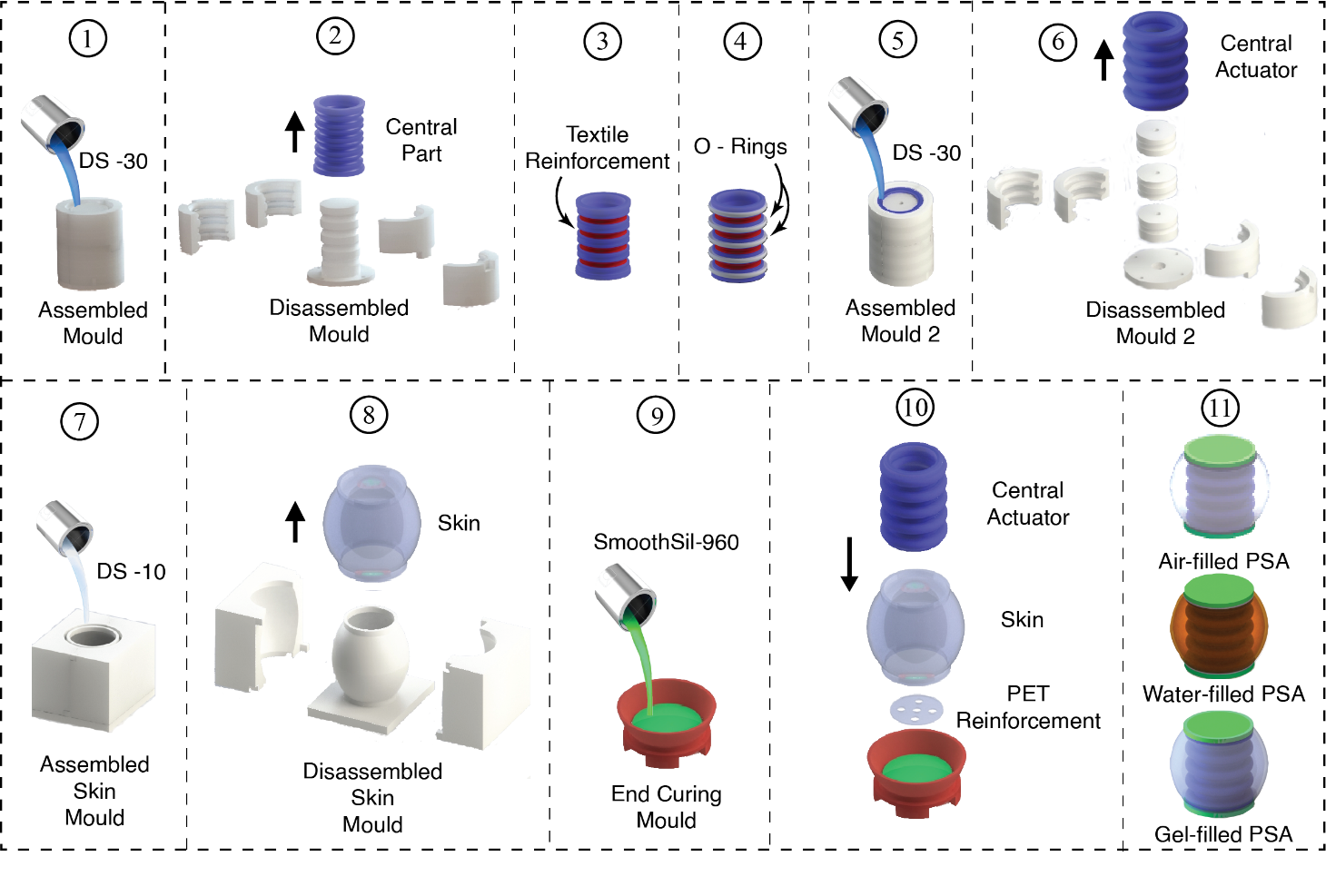
**Supplementary Figure S10:** Fabrication Steps of PSA (i) DS-30 poured into central part mould (ii) Mould dissembled to retrieve central part (iii) Textile reinforcement added in specific places painted with Silpoxy and acetone solution (iv) Layer of Silpoxy added on the inner side of O-rings to attach them to the central part (v) Central part put into another mould and an external layer of DS-30 added. (vi) Mould dissembled to take out the central actuator (vii) DS-10 poured into mould for elastomeric skin fabrication (viii) Cured skin retrieved dissembling mould (ix) SmoothSil-960 poured into end curing mould (x) After partial curing first PET sheet reinforcement placed then rest of the mould filled with SmoothSIL-960 and finally both the elastomeric skin and Central Actuator placed for curing. Similar procedure followed for the opposite side with placement of two tubes, one for actuation and another for accessing encapsulated chamber (xi) Fully cured PSA filled up with different fluids of varying quatities to undergo characterization

**Force interaction in radial force measurement setup**

Compression of the actuator creates radial force which is translated to the loadcell by means of the connected strings as shown in the force diagram in Supplementary Fig. S9. We consider the radial force generated to be F_R_, the tension produced in the string to be T, the longitudinal force on the load cell is F_L_, angle made by the string with the top plate θ and the actual force experienced by loadcell F_loadcell_. From the force interactions the following equations can be formulated:

$Tsin\theta=F_{R}$………………..(1)

$Tcos\theta=F_{L}$………………..(2)

$F_{R}=F_{L}tan\theta$………………(3)

$F_{Loadcell}=6F_{L}$……………(4)

$F_{R}= F_{Loadcell}tan\theta/6$…….(5)

Equation (5) implies that the radial force generated during compression of the actuator is related to both the recorded force value in the loadcell and the angular deformation of the actuator as shown in force diagram. Since the radial force acts on the entire surface of the skin hence the strings are able to account for a component of the total force but due to uniform conditions before measurement effect of different fluids and depth can compared from the results.


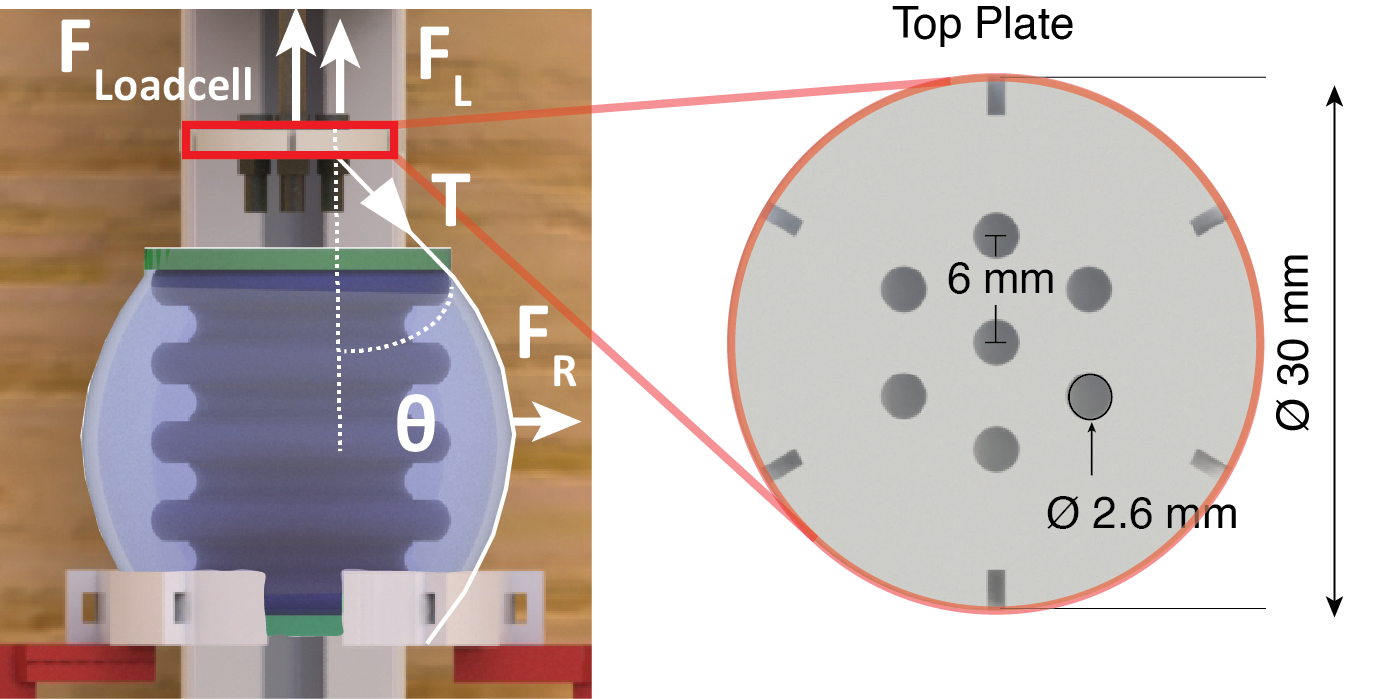


**Supplementary Figure S11**: Force Diagram of the actuator during radial force characterization illustrating the translation of the radial force (F_r_) via tensile force (T) through the string to the loadcell (F_loadcell_). Top plate with equidistant holes for passing strings ensured the cancellation of force components in the horizontal direction.

**Supplementary Video S1:**

This video explains the working principle of the actuator. The actuator with positive and negative pressure attains two antagonistic configurations from a neutral one, generating longitudinal and radial force respectively.

**Supplementary Video S2:**

This video illustrates how the presence of setae like friction pads enhance the locomotion performance of the robot on a planar surface (green mat) for both elongation gait pattern (EGP) and combined gait pattern (CGP).

**Supplementary Video S3:**

This video illustrates the effect of the presence of setae-like friction pads on the locomotion performance of the robot on granular medium surface for both elongation gait pattern (EGP) and combined gait pattern (CGP). The effect of the gait pattern for the same robot without setae at different depths (20 mm and 40 mm) has also been shown.

**Supplementary Video S4:**

This video illustrates the locomotion capability of the peristaltic soft robot within a confined environment like a pipe.

**Supplementary Video S5:**

This video illustrates the steps involved in the fabrication a Peristaltic Soft Actuator.
